# Solar Driven Gas Phase Advanced Oxidation Processes for Methane Removal ‐ Challenges and Perspectives

**DOI:** 10.1002/chem.202201984

**Published:** 2022-09-19

**Authors:** Jie Zhang, Yuyin Wang, Yun Wang, Yang Bai, Xin Feng, Jiahua Zhu, Xiaohua Lu, Liwen Mu, Tingzhen Ming, Renaud de Richter, Wei Li

**Affiliations:** ^1^ State Key Laboratory of Materials-Oriented Chemical Engineering College of Chemical Engineering Nanjing Tech University Nanjing 210009 P. R. China; ^2^ Institute for Materials and Processes School of Engineering The University of Edinburgh Edinburgh UK; ^3^ School of Civil Engineering and Architecture Wuhan University of Technology Wuhan 430070 P. R. China; ^4^ Tour-Solaire.fr, 8 Impasse des Papillons 34090 Montpellier France

**Keywords:** advanced oxidation process, chlorine, methane oxidation, ozone, photocatalysis, photoreactor

## Abstract

Methane (CH_4_) is a potent greenhouse gas and the second highest contributor to global warming. CH_4_ emissions are still growing at an alarmingly high pace. To limit global warming to 1.5 °C, one of the most effective strategies is to reduce rapidly the CH_4_ emissions by developing large‐scale methane removal methods. The purpose of this perspective paper is threefold. (1) To highlight the technology gap dealing with low concentration CH_4_ (at many emission sources and in the atmosphere). (2) To analyze the challenges and prospects of solar‐driven gas phase advanced oxidation processes for CH_4_ removal. And (3) to propose some ideas, which may help to develop solar‐driven gas phase advanced oxidation processes and make them deployable at a climate significant scale.

## Graphical abstract

**Methane** (CH_4_) is a potent greenhouse gas and the second highest contributor to global warming. Developing large‐scale methane removal methods is one of the most effective strategies to reduce rapidly the CH_4_ emissions and thus to limit global warming. Challenges and prospects of solar‐driven gas phase advanced oxidation processes for CH_4_ removal are analyzed.

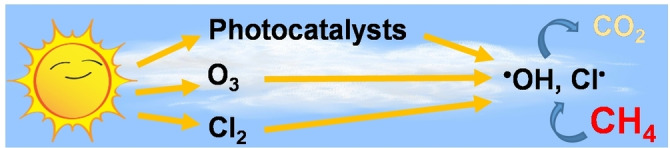

## 1 Introduction

### 1.1 Why focus on removing CH_4_ greenhouse gas?

Economic development is often accompanied by the sacrifice of the environment. Since the industrial revolution, human activities have produced a large amount of greenhouse gases (GHGs), thus resulting in the greenhouse effect. CH_4_ is a potent GHG. For a 100‐year time horizon, CH_4_ has a global warming potential (GWP) 27–35 times higher than that of CO_2_.^[1]^ It also has a short residence time in the atmosphere with a GWP 84 times higher than that of CO_2_ over 20 years.

**Figure 1:**
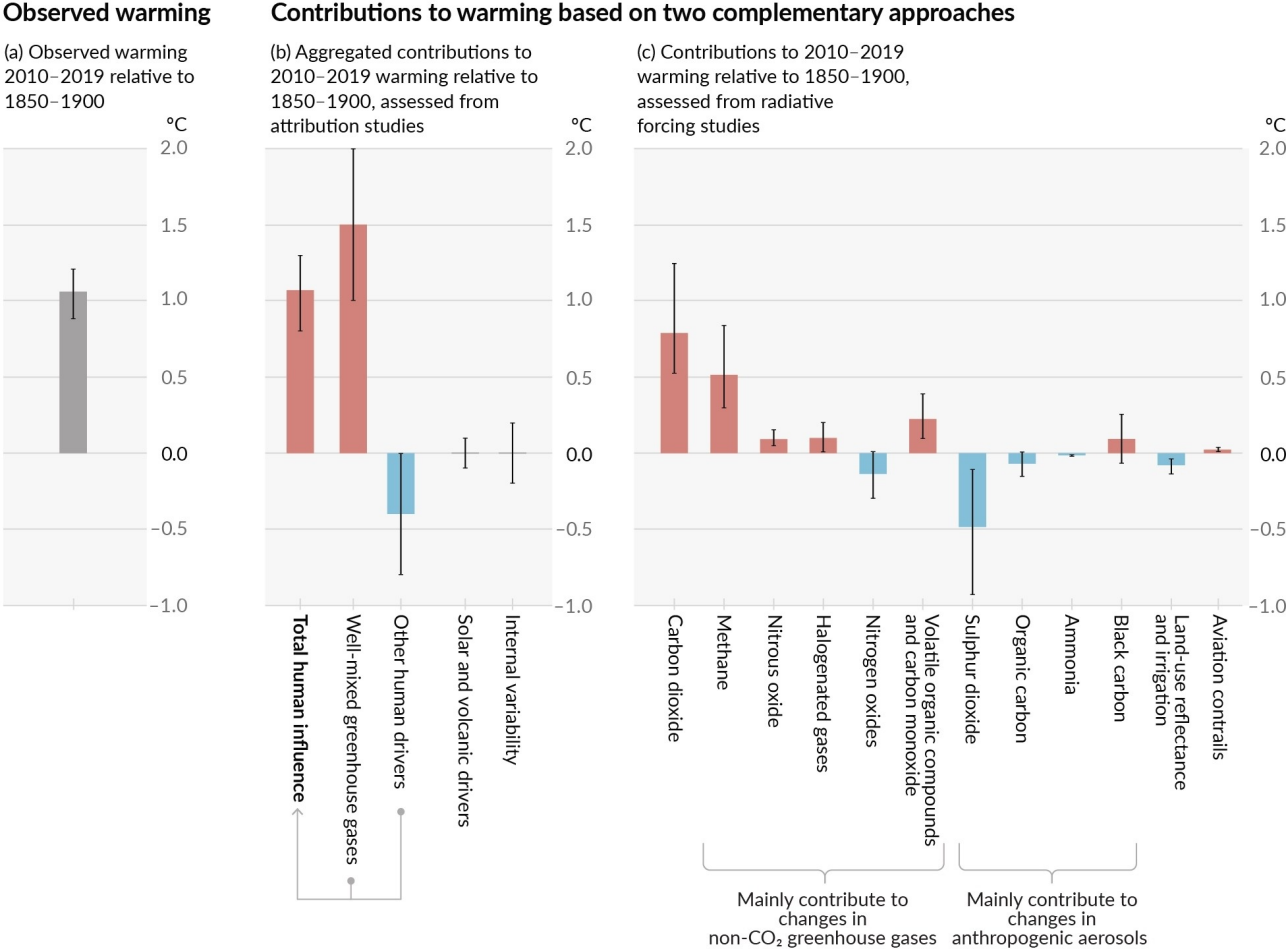
Assessed contributions to observed warming in 2010–2019 relative to 1850–1900. Reproduced with permission. Copyright 2021, IPCC.

At present, CO_2_ is the main cause of the greenhouse effect (about 0.75 °C global warming), while about 0.5 °C global warming is caused by CH_4_,^[2]^ as shown in Figure 1.

Apart from the attention and efforts that have been put towards CO_2_, it is more and more important and urgent to focus on CH_4_, too. We need to investigate in two directions ‐ CH_4_ emissions and CH_4_ already in the atmosphere.

In general, CH_4_ emission sources can be classified into two categories: natural sources of CH_4_ (∼40 %) and anthropogenic sources of CH_4_ (∼60 %). In November 2021, during the 26^th^ conference of parties (COP 26) in Glasgow, UK, more than 100 countries signed the global methane pledge committing to reduce anthropogenic methane emissions by 30 % comparatively to 2020 levels by 2030.

**Figure 2:**
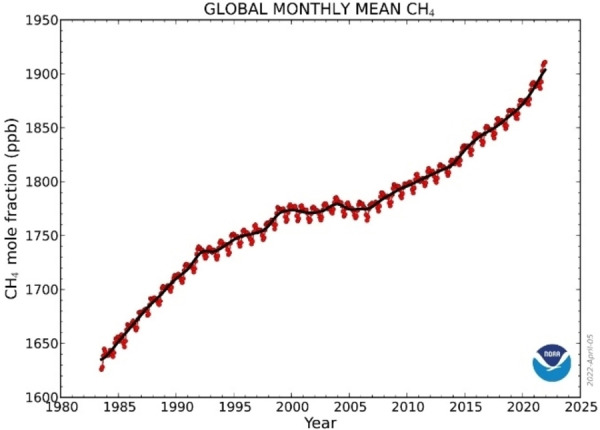
CH_4_ trend: This graph shows globally averaged, monthly mean atmospheric methane abundance determined from marine surface sites since 1983. Values for the last year are preliminary (NOAA Global Monitoring Laboratory).

Considering CH_4_ already in the atmosphere, its concentration averaged at 1,895.7 ppb during 2021, or around 162 % greater than pre‐industrial levels. Meanwhile, CH_4_ continues accumulating in the atmosphere at an alarmingly high rate. According to the US National Oceanic and Atmospheric Administration (NOAA), in 2020 and 2021 the annual increases in atmospheric methane (respectively 15.3 and 17 ppb) were the largest annual increases ever recorded since systematic measurements began, as shown in Figure 2.^[3]^

Limiting global warming below 1.5 °C as targeted by the Paris agreement will require not only stopping CO_2_ emissions but also reducing the emissions of CH_4_ ^[4]^ and removing CH_4_ from the atmosphere.^[5]^

### 1.2 Technology gap

CH_4_ is better known as a fuel or platform chemical, when its concentration is higher than 0.25 % (e. g., 2500 ppm), rather than a potent GHG. Mature technologies are available in this concentration range to utilize it as a fuel or platform chemical.

**Figure 3:**
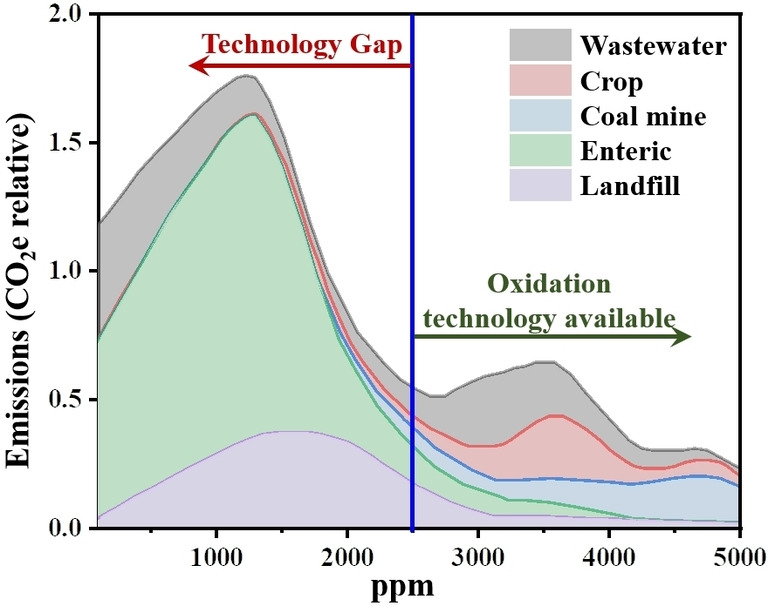
Relative amount of CH_4_ emissions at different concentrations from various emission sources.

However, the majority of CH_4_ emissions (e. g., from agriculture, landfill, and wastewater, as shown in Figure 3) are dilute and in concentrations lower than 2500 ppm. The concentration of CH_4_ in the atmosphere is even lower (i. e., 1895.7 ppb). No mature technology is available to utilize or remove CH_4_ in these concentrations.

Novel technologies are needed to fill this technology gap. Researchers are learning from the fate of CH_4_ in the natural atmosphere. Currently, in the troposphere, the principal natural CH_4_ sinks are hydroxyl radicals,^[6]^ chlorine atoms,^[7]^ minerals in soils and dust,^[8]^ soil microbes, plants, and trees. Enhancing or mimicking those natural sinks is the strategy of some early attempts to develop removal technologies for low‐concentration methane. For example, several advanced oxidation processes, which produce hydroxyl radicals and chlorine atoms, can transform CH_4_ into CO_2_, water vapor, and small amounts of volatile compounds, all of which are much less potent GHGs than the precursor. Relevant chemistry is summarized in Table 1. The basic principles of these conversions are well established and they happen naturally in the atmosphere.

**Table 1:** Main advanced oxidation processes to transform methane.

	Heterogeneous photocatalysis	Ozone based photochemistry	Chlorine based photochemistry
Main oxidizing reactive species	hydroxyl radical (⋅OH)	hydroxyl radical (⋅OH)	Chlorine atom (Cl)
Oxidation reactions of methane	CH_4_+O_2_+(⋅OH or Cl)→CO_2_+(H_2_O or HCl)		

As the vast majority of GHG removal work focuses on CO_2_, research into CH_4_ removal has been gaining momentum in recent years, particularly those based on the above‐mentioned advanced oxidation processes. There is early stage research (including conceptual proposals,^[9]^ numerical analysis,^[10]^ and experimental work^[11]^) spanning the areas of materials (e. g., TiO_2_ and Ag/ZnO photocatalysts,[^11a^, ^12^] zeolite catalyst^[9b]^), processes (e. g., Cl atoms generated from NaCl of natural sea‐spray aerosols^[13]^) and reaction systems (e. g., hydroxyl radical reactions and photolysis modules in ventilation systems^[14]^).

### 1.3 Solar driven gas phase advanced oxidation processes can be good options

We learned from CO_2_ removal that removing low concentration CO_2_ at a large scale is energy intensive.^[15]^ This applies to CH_4_ removal, too. Therefore, among those above‐mentioned advanced oxidation processes, the ones that can be driven by solar energy are more attractive.

**Figure 4:**
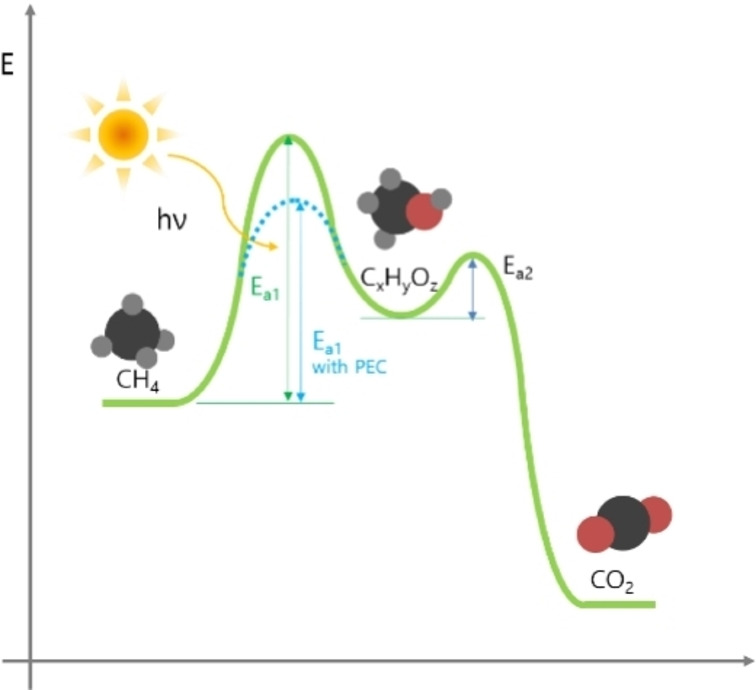
Schematic energy diagram of methane oxidation with a photocatalyst. Reproduced from Ref. [16] with permission from Catalysts, copyright 2021.

For example, photocatalysis is an ideal way to replace traditional thermal catalysis in some particular applications.^[17]^ It employs photons to drive chemical processes instead of thermal energy, and most importantly, photocatalysis enables difficult chemical reactions to occur at mild temperature conditions. Due to the chemical inertness of CH_4_ molecules, converting CH_4_ via thermal catalysis requires large activation energy. Photocatalysis reaction can generate high‐energy charge carriers in the process, which can pre‐activate CH_4_ and substantially reduce the activation energy.^[18]^ This pre‐activation process enables thermodynamically unfavorable reactions at room temperature, and overcomes traditional thermodynamic barriers (Figure 4).^[16]^ Compared with thermal catalysis, photocatalytic reactions can theoretically avoid harsh reaction conditions.

This perspective article focuses on solar driven advanced oxidation processes ‐ analyses their challenges, summarizes the state‐of‐the‐art progress, and proposes further solutions.

### 1.4 Challenges in solar driven gas phase advanced oxidation processes

There are hurdles to overcome in transforming these established principles and early concepts into validated technologies. Two crucial aspects are – (1) Efficacy. We need highly efficient photocatalysts or photochemical processes for the extremely diluted target gas. (2) Upscaling. The scale of any GHG removal technology needs to be significant to have a climate impact while the generation of airflow on a large scale is energy intensive.

In order to respond to all these challenges, this perspective article is organized in the following structure ‐ Section 2, Catalysts in heterogeneous photocatalysis for CH_4_ oxidation; Section 3, Processes of homogenous photochemistry for CH_4_ oxidation and Section 4, Photoreactors for large scale CH_4_ oxidation. Each section is started with the state‐of‐the‐art and is completed with future perspectives.

## 2 Catalysts in Heterogeneous Photocatalysis for CH_4_ Oxidation

**Figure 5:**
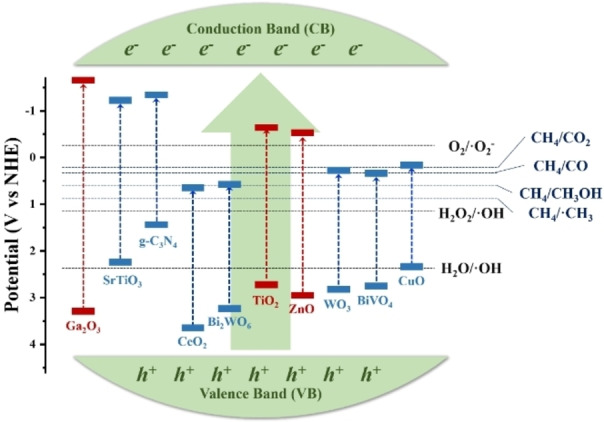
Positions of conduction band and valence band edges of various semiconductors and redox potentials of relevant chemical reactions, with respect to NHE.

Photocatalyst is a key part of a photocatalytic process, which refers to a kind of substance that can induce photocatalytic oxidation‐reduction reactions under light irradiation. The primary criterion for a suitable photocatalyst is that it fulfills the thermodynamic requirements of CH_4_ oxidation. The common and widely accepted way to present and discuss thermodynamic requirements for photocatalysis is a diagram (as shown in Figure 5) that contains two sets of information: 1) the band structure of electronic energy in photocatalysts, and 2) the redox potentials of the relevant chemical reactions.[^18b^, ^19^]

The positions of redox potentials of the relevant chemical reactions indicate their thermodynamic requirements. The redox potentials of oxidizing CH_4_ to different products are *E* ^*o*^ (CH_4_/CO_2_)=0.17 V, *E* ^*o*^ (CH_4_/CO)=0.32 V, *E* ^*o*^ (CH_4_/CH_3_OH)=0.65 V, and *E* ^*o*^ (CH_4_/CH_3_)=0.83 V, versus the normal hydrogen electrode (NHE). The oxidation of CH_4_ is initiated by radicals that attack the C−H bond. The redox potentials of commonly involved radicals are *E* ^*o*^ (O_2_/O_2_^−^)=−0.33 V, *E* ^*o*^ (H_2_O_2_/OH)=1.07 V, and *E* ^*o*^ (H_2_O/OH)=2.30 V, vs. NHE.^[18]^

The positions of conduction band (CB) and valence band (VB) edges of various semiconductors indicate their thermodynamic capability to generate active radicals and drive a chemical reaction.^[20]^ In this case, the CB of the semiconductor is thermodynamically required to be located higher than the reduction potential of O_2_/O_2_, while the VB needs to be located below the oxidation potentials of H_2_O_2_/OH and H_2_O/OH.^[21]^

Therefore, Figure 5 has clearly displayed the thermodynamic requirements for methane oxidation and the thermodynamic capabilities of various photocatalysts. The most suitable photocatalysts for photocatalytic oxidation of CH_4_ are TiO_2_, ZnO, and Ga_2_O_3_. Recent progress in the field agrees with this and the best results mostly came from photocatalysts based on TiO_2_, ZnO, and Ga_2_O_3_.

### 2.1 TiO_2_

Since Japanese scientists Fujishima and Honda discovered that TiO_2_ single crystal electrodes were able to split water in 1972, systematic research on TiO_2_ photocatalysis has kicked off.^[22]^ TiO_2_ is the most widely used semiconductor photocatalyst, which is chemically stable, environmentally friendly, and inexpensive.

The vast majority of current work on TiO_2_ photocatalyst for CH_4_ oxidation is in the field of CH_4_ conversion,^[22]^ where CH_4_ is seen as a platform chemical and appears in high concentrations. The emphasis in this area is on selectivity and avoiding overoxidation to CO_2_.^[22]^ This makes the research results not directly applicable for oxidation of low concentration CH_4_ to CO_2_, but can be inspiring as in some cases there are in‐depth discussions into some catalysts and their mechanism of over‐oxidation. For example, Song et al.^[23]^ demonstrated that a platinum decorated TiO_2_ photocatalyst was a ‘bad’ sample because of heavy over‐oxidation, but this can be a ‘good’ starting point for a different application (i. e., total oxidation of low concentration CH_4_).

There are scattering of publications in the last decade working towards total oxidation of low concentration CH_4_ on TiO_2_. Kleinschmidt and Haeger et al. investigated the kinetics of the oxidation reactions from CH_4_ to fully oxidized CO_2_, in a concentration range from 2000 to 15000 ppm.^[24]^

Jin et al. evaluated the feasibility of photocatalytic oxidation of ventilation air CH_4_ for coal mining fugitive emissions abatement. Results showed that the simulated ventilation air (with CH_4_ concentration in the range from hundreds of ppm to 3000 ppm) can be oxidized at ambient temperature by photocatalytic reaction with commercial TiO_2_, however, the reaction rate was slow.^[25]^

To the best of our knowledge, there is no reported research on the photocatalytic oxidation of ∼2 ppm atmospheric methane. We made the first attempt recently. A flow through photocatalytic reactor was developed to test ∼2 ppm atmospheric CH_4_ in a continuous flow mode, using commercial TiO_2_ (P25) as the photocatalyst illuminated by 20 W/m^2^ UV light (similar to UV intensity in solar radiation).

**Figure 6:**
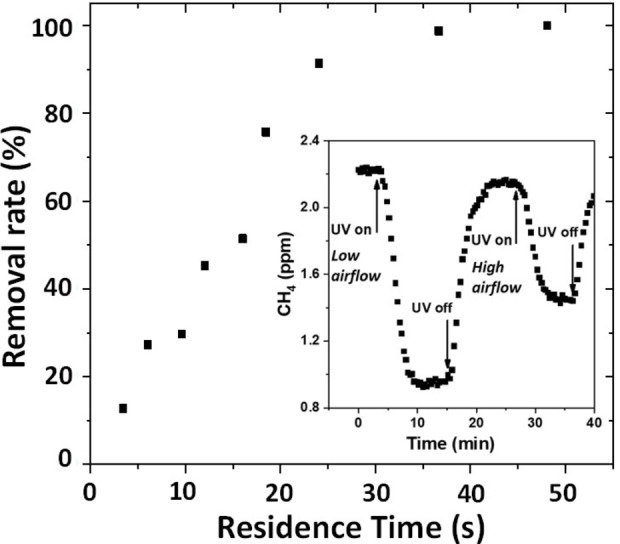
Photocatalytic removal of ∼2 ppm atmospheric CH_4_ in a bench top continuous flow reaction system.

As shown in the inset of Figure 6, under an airflow of 0.4 L/min, which is equivalent to a residence time of 12 seconds, the removal rate of CH_4_ is 46 %. Faster airflows lead to shorter residence time and give lower removal rates. Slower airflows result in longer residence time and provide higher removal rates, which can be as high as 98 % when residence time is longer than half a minute (Figure 6).

### 2.2 ZnO

In the field of photocatalytic conversion of CH_4_, where CH_4_ is seen as a platform chemical and appears in high concentrations, ZnO has also emerged as a promising photocatalyst.^[26]^ It shows many advantages such as suitable optical band structure and electronic properties. ZnO has three different crystalline forms, namely wurtzite, zinc blende, and rock salt structures, among which the wurtzite ZnO crystal structure (the most common because of its high thermodynamic stability) appears most often in CH_4_ conversion.

**Figure 7:**
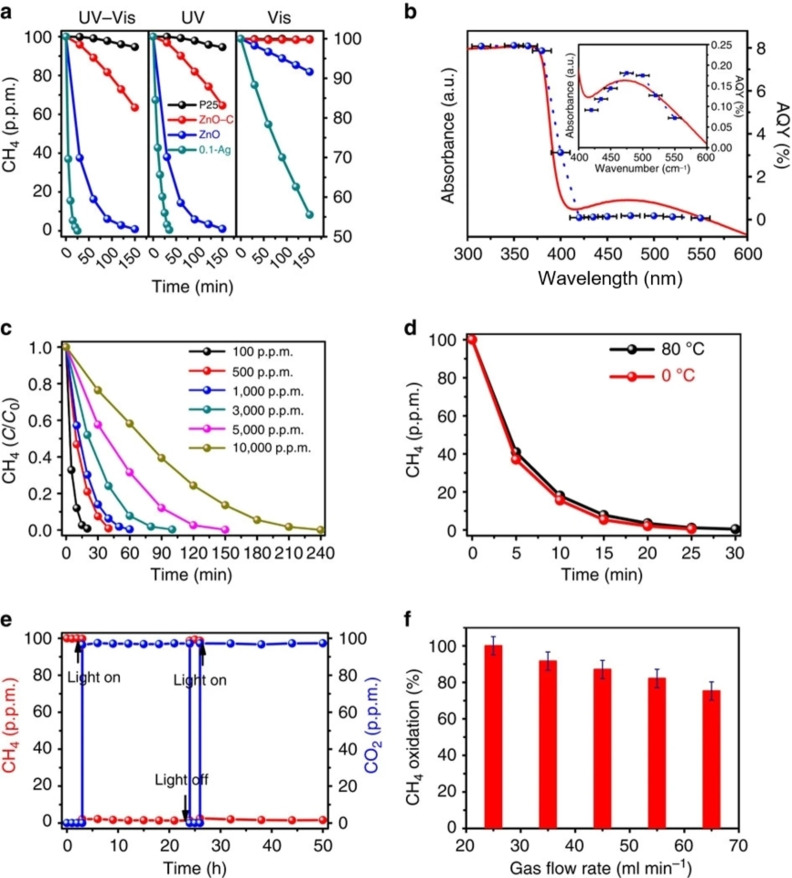
(a) Photocatalytic oxidation of methane in a fixed‐bed mode with full arc (UV‐vis), ultraviolet and visible light illumination, respectively. (b) Ultraviolet‐visible diffuse reflectance spectrum and AQYs of the 0.1‐Ag sample plotted as a function of wavelength of the incident light. (c) Time evolution of the methane photo‐oxidation over the 0.1‐Ag sample in the fixed‐bed mode under full arc illumination with various initial CH_4_ concentration. (d) Influence of the temperature on the methane photo‐oxidation activities over the 0.1‐Ag sample under full arc illumination. (e) Methane photo‐oxidation activity over the 0.1‐Ag sample under full arc illumination and a flow‐gas mode with gas flow rate of 25 ml min^−1^. (f) Influence of the gas flow rate on the rate of methane oxidation under the flow‐gas mode with ±5 % error bars calculated from the sample introduction uncertainty. Reproduced from Ref. [11a] with permission from Nature Communications, copyright 2016.

Chen Yi et al.^[11a]^ extended the research into photocatalytic oxidation of CH_4_ in much lower concentrations (100∼10000 ppm). In their study, as shown in Figure 7, 200–300 nm sized commercial ZnO can already oxidize a considerable amount of CH_4_ at a concentration of 100 ppm. They demonstrated that by reducing their size to ∼20 nm, ZnO nanoparticles exhibited high activity for methane oxidation under simulated sunlight illumination, and a small amount (0.1 wt %) of nano silver decoration further enhances the photocatalytic oxidation of CH_4_ via the surface plasmon resonance. They achieved a quantum yield of 8 % at wavelengths <400 nm and over 0.1 % at wavelengths ∼470 nm on the silver decorated ZnO nanoparticles.

In addition to the batch‐wise test in a fixed‐bed mode, they also tested photocatalytic oxidation of CH_4_ in a continuous flow mode, which is even more relevant to future practical applications. For 100 ppm CH_4_, 1–2 seconds of residence time is sufficient to achieve >80 % removal rate.

The CH_4_ concentrations they tested are in the range of 100 to 10000 ppm, and fit well with the concentration range where the technology gap is. This shows great promise for CH_4_ GHG removal.

### 2.3 Hybrid photocatalysts

Both TiO_2_ and ZnO meet most of the criteria for an ideal photocatalyst, but there are weak points, such as limitation to ultraviolet light and recombination of charge carriers.[^20b^, ^27^] Introducing co‐catalysts or heterojunctions to form hybrid photocatalysts is a promising strategy to overcome these weaknesses.^[28]^ The primary criterion for a suitable hybrid photocatalyst is the same as for a single semiconductor (i. e., it needs to fulfill the thermodynamic requirements of CH_4_ oxidation).

**Figure 8:**
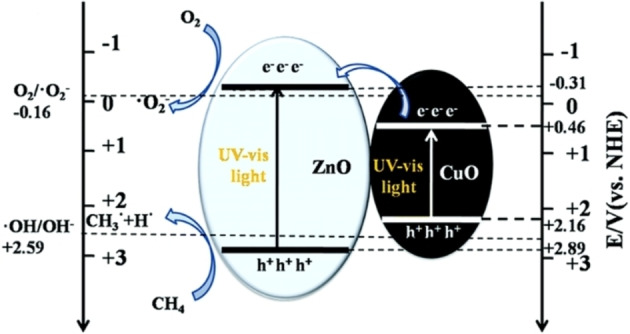
Schematic of the cocatalytic function of CuO on CH_4_ oxidation over ZnO under simulated solar light illumination. Reproduced from Ref. [29] with permission from Journal of materials chemistry A, copyright 2019.

Li et al.^[29]^ successfully synthesized a CuO/ZnO photocatalyst for total oxidation of CH_4_, as shown in Figure 8. The band edge potential of CuO is not suitable to activate the O_2_, but it has a narrow bandgap of 1.7 eV, so the CuO/ZnO composite can absorb much more solar light. Part of *e* ^*−*^ in the CB of CuO can be excited to the CB of ZnO, thereby activating the oxygen molecular to generate free radicals.

**Figure 9:**
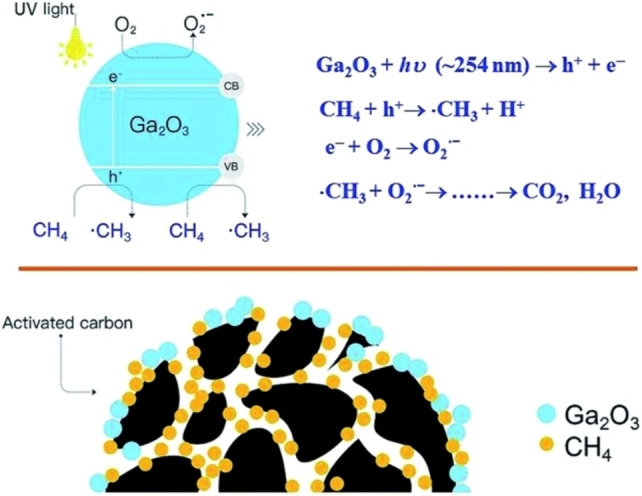
Proposed mechanism for photocatalytic oxidation of CH_4_ over Ga_2_O_3_/AC composites. Reproduced from Ref. [30] with permission from RSC advances, copyright 2017.

Wei et al.^[30]^ prepared β‐Ga_2_O_3_ supported on activated carbon (AC) composites for efficient photocatalytic oxidation of CH_4_ to CO_2_ under UV irradiation, as shown in Figure 9. Among them, AC can enhance the adsorption of CH_4_ molecules, thereby transferring CH_4_ to the catalytic active component β‐Ga_2_O_3_. β‐Ga_2_O_3_ has a wide bandgap that meets the requirements for strong oxidizing ability and effectively promotes the separation of photogenerated electron‐hole pairs. The synergistic effect improves photocatalytic performance.

### 2.4 The prospect of photocatalysts for low‐concentration CH_4_

Learned from the above existing research and the fact that CH_4_ GHG is often extremely diluted (e. g., <2500 ppm), the following aspects could be the major topics for future development on photocatalysts for this application.

High surface area is always a favorable feature for an ideal photocatalyst and this is more desirable to dealing with low‐concentration CH_4_. Nano‐engineered or mesoporous photocatalysts can be good options. In the authors’ research group, more than ten years of research experience has been accumulated in the area of nano‐engineering mesoporous TiO_2_.^[31]^ It is thermally stable, highly crystalline, reactive, and has a large surface area,^[32]^ which are all desirable properties as a photocatalyst alone or as a support for more advanced hybrid photocatalysts. Ongoing research is focusing on hybrid photocatalysts based on this mesoporous TiO_2_. Another option is that photocatalysts can be loaded to high surface area supports, for example, porous frameworks (MCM‐41^[33]^), and glass fiber mesh/cloth.^[34]^

Enhancing CH_4_ adsorption would be another strategy to improve the oxidation of low‐concentration CH_4_. The activated carbon (AC) supported β‐Ga_2_O_3_ composite^[30]^ mentioned earlier is a good example. Surface modification of photocatalysts to increase the affinity with CH_4_ could be another possible way to enhance CH_4_ adsorption.

Visible light utilization is the key to make photocatalysis more applicable. It is one of the major goals that researchers in the field are pursuing. Solar driven photocatalysis for CH_4_ GHG removal relies even more on visible light utilization. The encouraging thing is that the cutting‐edge progress in this direction is developing so rapidly. Any exciting progress made by someone can inspire the development of visible light photocatalysts for CH_4_ GHG removal.

As shown in the above examples (i. e. Ag/ZnO, CuO/ZnO, and Ga_2_O_3_/AC), hybrid photocatalysts can be designed to address every challenge discussed above. For example, efficient photocatalysts can be loaded and spread into porous and high surface area substrates to increase photocatalytic surface and enhance CH_4_ adsorption; different semiconductors can be utilized to construct heterojunctions to fulfill the thermodynamic requirements for methane oxidation and to expand the visible light utilization; co‐catalysts can be combined with semiconductors to boost adsorption and reaction.

## 3 Processes of Homogenous Photochemistry for CH_4_ Oxidation

As mentioned earlier, researchers are learning from the natural CH_4_ sinks in the troposphere, for example, hydroxyl radicals and chlorine atoms (as summarized in Table 1). These are all homogenous photochemistry processes. Mimicking those natural sinks is also a strategy to develop removal technologies for low concentration CH_4_.

### 3.1 Ozone (O_3_) photochemistry

Ozone is a powerful oxidant capable of reacting with a wide range of organic and inorganic compounds. It has a higher oxidation potential (*E°*=2.08 eV), second only to fluorine (*E°*=2.87 eV) and hydroxyl radicals (*E°*=2.30 eV). Since ozone can absorb ultraviolet light of 200∼400 nm, its photolysis can promote the generation of highly active substances such as O⋅ and ⋅OH, which can improve the pollutants removal and mineralization.^[35]^

During photocatalytic oxidation, the formation pathway of hydroxyl radicals is described in Equation (1). In the case of ozone photolysis, oxidants [e. g., O⋅, Equation (2)] will be generated. Ozone can react with H_2_O to generate hydroxyl radicals through electron‐hole pairs, light irradiation, or active sites on catalysts^[36]^ [Equation (3)]. Ozone decomposition is primarily carried out by the following five‐step chain reaction, as shown in Equations (4‐7.^[37]^

(1) $$3e{}^{-}+3\;h{}^{+}+2H{}_{2}O+O{}_{2}\;\to 4{}^{•}\mathrm{OH}$$

(2) $$O{}_{3}\to {}^{•}O+O{}_{2}$$

(3) $$O{}_{3}+H{}_{2}O\to 2{}^{•}\mathrm{OH}+O{}_{2}$$

(4) $$O{}_{3}+\mathrm{OH}{}^{-}\to {}^{•}O{}_{2}{}^{-}+{}^{•}\mathrm{HO}{}_{2}$$

(5) $$O{}_{3}+{}^{•}\mathrm{OH}\to O{}_{2}+{}^{•}\mathrm{HO}{}_{2}↔{}^{•}O{}_{2}{}^{-}+H{}^{+}$$

(6) $$O{}_{3}+{}^{•}\mathrm{HO}{}_{2}\overset{\leftarrow }{\to }2O{}_{2}+{}^{•}\mathrm{OH}$$

(7) $$2{}^{•}\mathrm{HO}{}_{2}\to O{}_{2}+H{}_{2}O{}_{2}$$

**Figure 10:**
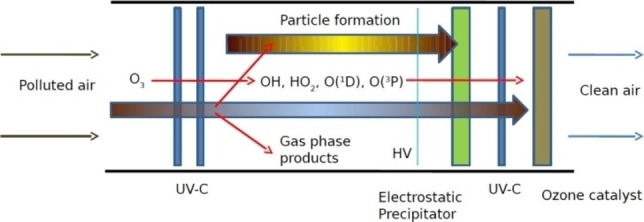
Photochemical air purification process used in the prototypes. Ozone is added to the airstream, and HO_x_ radicals are generated with UV‐C lamps. Particles form and are removed by the electrostatic precipitator. Gas‐phase products are removed in a second stage of radical chemistry. Finally, excess ozone is removed using a catalyst. Reproduced from Ref. [14] with permission from Environmental Science & Technology, copyright 2014.

To the best of our knowledge, there is no existing publication that demonstrates the removal of low concentration CH_4_ using ozone photolysis. Several most relevant studies are presented here. Li et al. and Jin et al. studied CH_4_ oxidation using ozone with the assistance of either thermal catalysis or solid catalysts.^[38]^ Johnson et al. demonstrated a gas‐phase advanced oxidation process. They used ozone and UV‐C light to produce in situ radicals to oxidize air pollution (e. g., propane, cyclohexane, benzene, isoprene, aerosol particle mass), as shown in Figure 10.^[14]^ This process and set‐up can be adapted to investigate the removal of low concentration CH_4_ using ozone photolysis.

### 3.2 Chlorine (Cl⋅) photochemistry

In the troposphere, chlorine atoms (Cl⋅) are also an important oxidant,^[39]^ about 2.5 % of the total CH_4_ is oxidized by Cl⋅.^[40]^ In some polluted coastal areas, Cl⋅ accounts for ∼10 to >20 % of total marine boundary layer CH_4_ oxidation.^[41]^ Although the ⋅OH oxidizes about 90 % of CH_4_, it is interesting to target the Cl⋅ sink because the speed of CH_4_ reaction with Cl⋅ is 16 times faster than the one of CH_4_ with ⋅OH.^[42]^ The rate constant of the reaction between CH_4_ and Cl⋅ is 1.07×10^−13^ cm^3^ s^−1^,^[43]^ while the rate constant of the reaction between CH_4_ and hydroxyl radicals is 6.20×10^−15^ cm^3^ s^−1^.^[44]^

Polat et al. demonstrated a photochemical method for efficient removal of ∼2 ppm CH_4_ via Cl⋅ initiated oxidation. The following reactions outline the mechanism of Cl⋅ induced CH_4_ oxidation technology.^[11b]^

(8) $$\mathrm{Cl}{}_{2}+hv\to 2\mathrm{Cl}{}^{•}$$

(9) $$\mathrm{Cl}{}^{•}+\mathrm{CH}{}_{4}\to \mathrm{CH}{}_{3}+\mathrm{HCl}$$

(10) $$\mathrm{CH}{}_{3}+O{}_{2}+M\to \mathrm{CH}{}_{3}O+M$$

(11) $$\mathrm{CH}{}_{3}O+\mathrm{Cl}{}^{•}\to \mathrm{CH}{}_{3}O+\mathrm{ClO}$$

(12) $$\mathrm{CH}{}_{3}O+O{}_{2}\to \mathrm{HCHO}\;+\mathrm{HO}{}_{2}$$

(13) $$\mathrm{HCHO}+\mathrm{Cl}{}^{•}+O{}_{2}\to \mathrm{CO}+\mathrm{HCl}+\mathrm{HO}{}_{2}$$

### 3.3 The prospect of homogeneous photochemistry for low‐concentration CH_4_

Compared to heterogeneous photocatalysis, homogeneous photochemistry processes have the advantage of avoiding several potential rate‐limiting steps (e. g., gas diffusion towards the solid surface, adsorption and desorption at the gas‐solid interface, and surface reactions). This advantage can be more profound when we consider lower concentrations of target gas (e. g., low concentration CH_4_ in this context). Therefore, homogeneous photochemistry processes should be more advantageous for large scale applications of low concentration CH_4_ GHG removal. The next question is how we can produce a large amount of ozone or chlorine. Here we take chlorine as an example, as it reacts much faster with CH_4_ than ozone.

**Figure 11:**
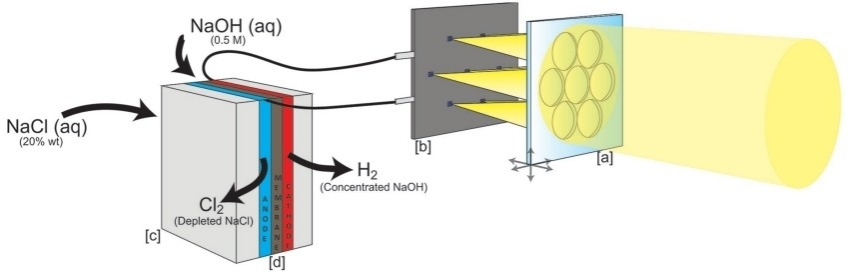
Solar chlor‐alkali device overview. a) Optical planar concentrator, lenses array. b) Triple‐junction Gas‐based solar cells mounted on PCB. c) Electrolyzer 3D printed metallic flow plates. d) Electrochemical cell, composed of a nickel cathode and a DSA insoluble anode, separated by a cation exchange membrane. Reproduced from Ref. [45] with permission from Global Challenges, copyright 2017.

It is possible to generate Cl⋅ by UV photolysis of chlorine gas (Cl_2_), which is produced at a large scale by the chlor‐alkali processes (e. g., electrolysis of sodium chloride salt (NaCl) aqueous solutions, or electrolysis of melted NaCl). These processes can all be integrated with solar cells to utilize solar energy, as shown in Figure 11.^[45]^

It is also possible to generate a large amount of Cl⋅ from Iron (III)/Iron (II) photo‐catalyzed reaction by UV at 365 nm, a natural catalytic process.^[46]^ It involves the illumination of an acidified aqueous solution of Fe (III), which induces a photochemical reduction of Fe (III) to Fe (II) and the formation of Cl⋅. Under the marine boundary layer, the Fe (II) species formed are re‐oxidized and FeCl_3_ is rapidly regenerated, making a profit from abundant sea brine which contains NaCl and provides excess chloride ions.^[47]^ Wittmer et al. demonstrated photochemical activation of Cl⋅ by dissolved iron in artificial sea‐salt aerosol droplets or by highly dispersed iron oxide (Fe_2_O_3_) aerosol particles exposed to gaseous HCl.^[13]^

## 4 Photoreactors for Large Scale CH_4_ Removal

In order to demonstrate the feasibility of solar‐driven gas phase advanced oxidation processes, the design of photoreactors is an equally critical area. It can scale up the laboratory tests and enable industrial applications.

### 4.1 Photoreactors at CH_4_ emission sources where there are defined outlets

There are two areas of tackling CH_4_ GHG, for example, reducing the emissions of CH_4_ at their sources and removing atmospheric CH_4_ from the open air. The methodologies in these two areas are different because of the different scenarios.

When there are defined outlets available, many existing designs of various gas phase photoreactors are applicable, for example, solar‐driven tubular reactor to treat polluted airstreams,^[48]^ and internal‐illuminated monolith photoreactor.^[49]^ There are comprehensive review articles available^[50]^ and any new progress in the field can feed into the development of photoreactors for applications at CH_4_ emission sources.

### 4.2 Photoreactors for large scale CH_4_ removal in the open air

In the open air, sufficient airflow is required to process extremely dilute CH_4_. de Richter et al. proposed a method to perform large scale CH_4_ oxidation using a solar chimney as an air moving device and coupling it with photocatalytic and/or photochemical processes.^[9a]^

**Figure 12:**
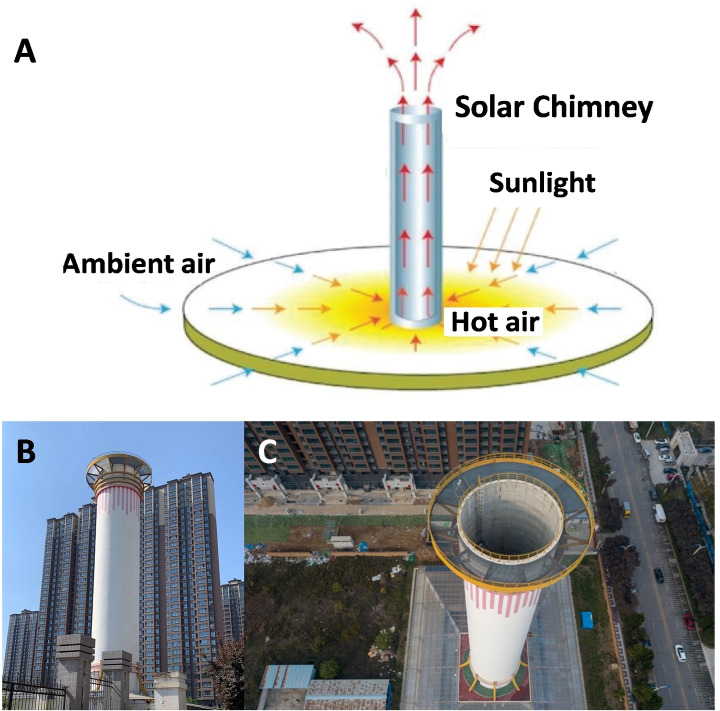
A) A scheme of the working principle of solar chimney. B) and C) Photographs of the SC prototype currently visible in the city of Xi'an, China. Pictures taken by one of the co‐authors.

A solar chimney (SC, as shown in Figure 12A) involves, a) a large greenhouse used as a solar collector where the air is heated to create an artificial updraft; b) a tall tower or chimney in the centre of the greenhouse to build up the stack effect.[^9a^, ^51^] One SC prototype has been built in 2018 in the city of Xi'an in China,^[52]^ with the purpose of air purification as shown in Figures 12B and 12 C.

The solar collector and the chimney can be much larger and taller than this prototype. Then turbines can be added at the bottom of the chimney to generate electricity. This makes it a solar chimney power plant (SCPP). A prototype SCPP with a 46,760 m^2^ solar collector and a 195 m high chimney was built and fully tested in 1982–1989^[53]^ in Manzanares, Spain.

**Figure 13:**
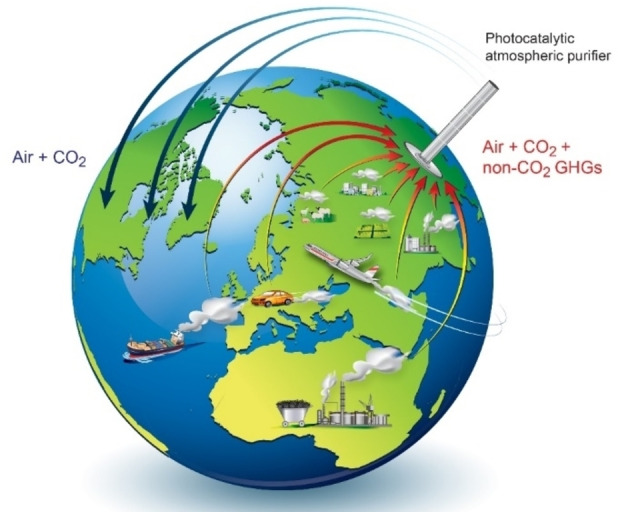
Artist's representation of the GHG removal technologies by photocatalytic‐SCs. Reproduced from Ref. [9a] with permission from Progress in Energy and Combustion Science, copyright 2017.

For both SC and SCPP, photocatalytic and/or photochemical processes can be coupled under the solar collector and/or in the chimney, where a large amount of airflow can be processed, as shown in Figure 13.^[9a]^

**Figure 14:**
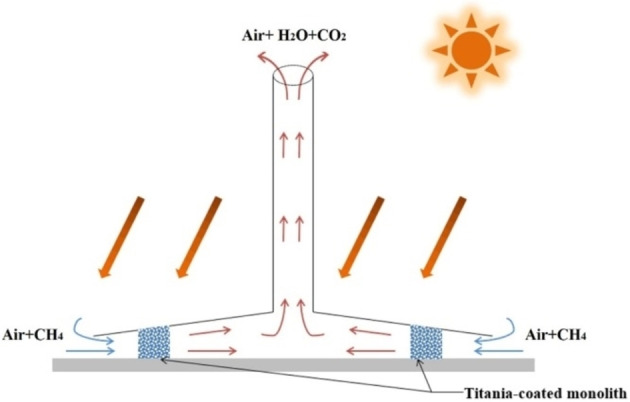
Solar chimney power plant integrated with a photocatalytic reactor (SCPP‐PCR) for atmospheric methane removal. Reproduced from Ref. [10a] with permission from Solar Energy, copyright 2021.

Ming et al. used computational fluid dynamics (CFD) models to investigate the performance and influencing factors of photocatalytic oxidation of CH_4_ under SCPP‐photocatalytic reactor (PCR) system,^[10a]^ as shown in Figure 14. The PCR was designed based on a honeycomb monolithic photoreactor. The flow characteristics of the system under different PCR dimensions were also analyzed, in which the pore diameter and length of the PCR had the largest effects on flow performance, including pressure drop, flow velocity and volume flow rate. When the channel diameter of the honeycomb PCR was 4 mm and its length is 8 m, the SCPP‐PCR system can remove 21,312 g CH_4_ per day.

Huang et al.^[10b]^ evaluated the feasibility of an SCPP‐PCR as a large‐scale photocatalytic reactor for removing atmospheric CH_4_. They calculated the potential of CH_4_ removal in relation to the dimensions and configuration of SCPP and different types of photocatalysts (e. g., TiO_2_, Ag‐doped ZnO). Night operation strategies and further improvements were also discussed.

### 4.3 The prospect of photoreactors development

**Figure 15:**
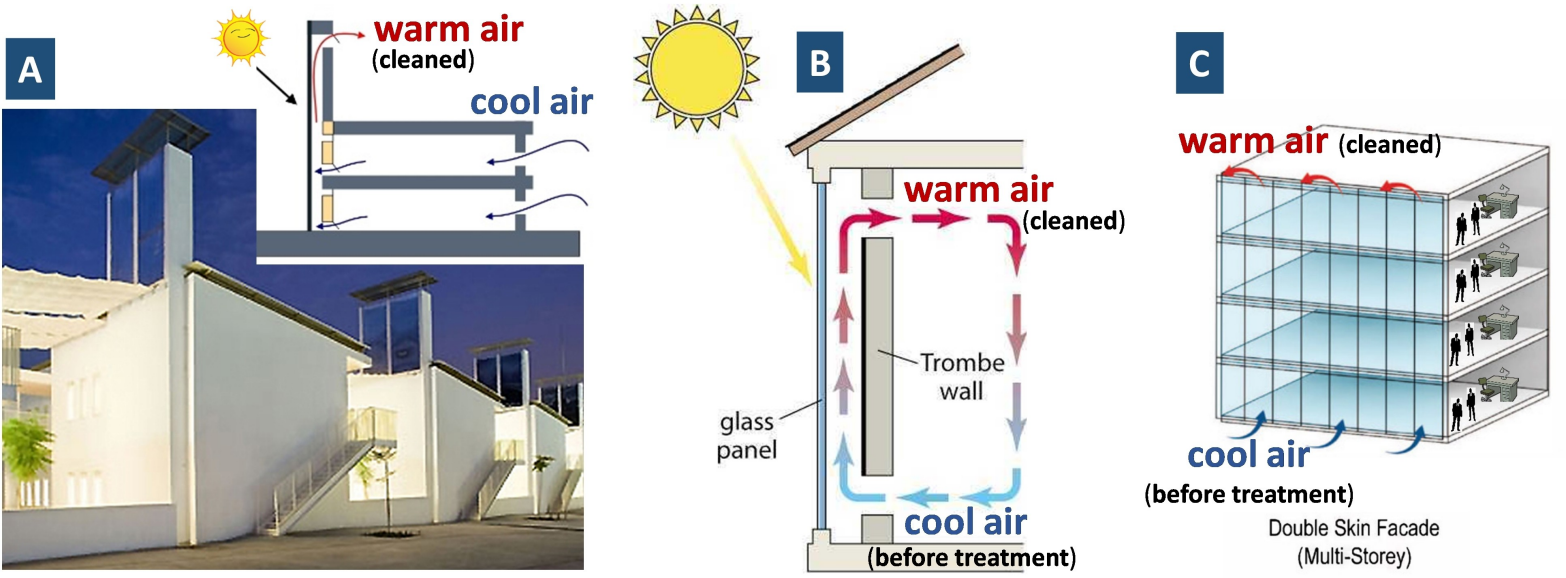
Other three formats of solar updraft: A) ventilation solar chimney, B) Trombe wall, and C) double skin facade.

Capital investment and land footprint could be some potential hurdles before giant SC/SCPP can be widely applied. With the same principle as SC, there are other three formats of solar updraft devices (namely Trombe wall, double skin façade, and ventilation solar chimney, Figure 15). When combined with advanced oxidation processes, they can provide more versatile applications for the above‐mentioned two scenarios (e. g., at CH_4_ emission sources and in the open air).

**Figure 16:**
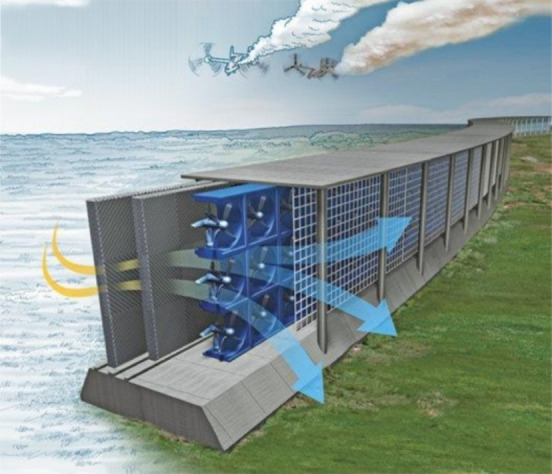
A hypothetical industrial DAC device with added photocatalyst, proposed to oxidize CH_4_. Reproduced from John Bradley.^[55]^

In the future development of global GHG removal, direct air capture (DAC) systems may play an important role. Once DAC plants exist, we would have a large amount of airflow. Therefore, de Richter et al. proposed that, to profit from this existing infrastructure, CH_4_ removal processes could be integrated to enhance the capture/removal of GHGs, as shown in Figure 16.^[54]^ Progresses in the fields of heterogeneous photocatalysts, homogeneous photochemical processes and photoreactors will all be critical to making this strategy feasible.

There are other nature mimicking ideas^[5b]^ that may be inspiring for researchers to design other photoreaction systems, which can be deployed at a large scale.

## 5 Conclusion

In this perspective, we focus on heterogeneous photocatalysts, homogeneous photochemical processes, and photoreactors for CH_4_ greenhouse gas removal. By summarizing some recent progress and future prospects, we try to inspire some solutions to fill the technology gap of removing low concentration (<2500 ppm) CH_4_, which appears in the atmosphere and from a wide range of CH_4_ emissions.

The achievements so far include conceptual proposals, numerical analysis, and early stage experimental work, spanning the areas of photocatalysts, photochemical processes, and reaction systems.

The fundamental challenge is that any GHG removal technology needs to be deployed at climate relevant scale. Therefore, it is important that solar driven gas phase advanced oxidation processes can remove low concentration methane at a large scale. This requires cheap and efficient photocatalysts, as well as cheap and large reaction systems.

It is positive to envisage that there are potential solutions. The cutting‐edge progress in newer and better photocatalysts is developing so rapidly thanks to the large photocatalysis research community. Any exciting progress can be adapted to improve CH_4_ GHG removal. For homogeneous photochemical processes, there is a lot to learn from the natural hydroxyl radical and chlorine sinks of CH_4_ happening all the time in the atmosphere. To make any solar‐driven gas phase advanced oxidation process deployable at a climate significant scale, big ideas and collective efforts are needed.

## Conflict of interest

The authors declare no conflict of interest.

6

## Biographical Information

*Jie Zhang graduated from Weifang University of Science and Technology in 2020 with a Bachelor's degree. She is currently a master candidate at Nanjing Tech University under the supervision of Prof. Liwen Mu and Prof. Xin Feng. Her research focuses on the photocatalysis and the high‐value utilization of biomass materials*.

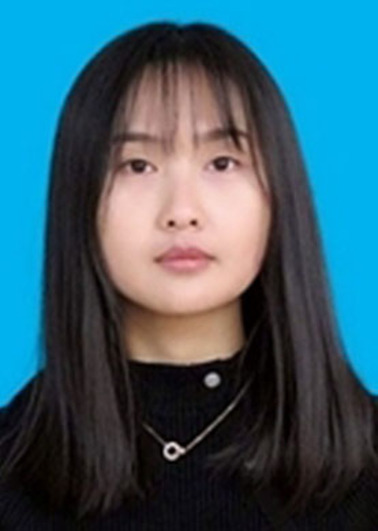

## Biographical Information

*Yuyin Wang received her B.Sc. degree from YangZhou University of in 2020. Currently, she is studying for a Ph.D. under the supervision of Dr Wei Li in the University of Edinburgh. Her research mainly focuses on catalytic processes and materials for methane oxidation*.

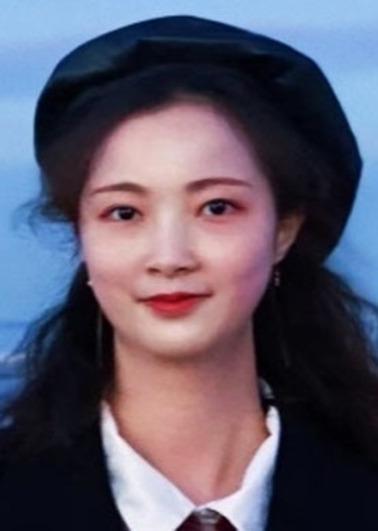

## Biographical Information

*Yun Wang, majoring in chemical engineering and technology, completed his bachelor's degree in 2021 at Weifang University of Science and Technology. In the same year, he joined Prof Xiaohua Lu's research group at in Nanjing Tech University and is currently focusing on photocatalytic removal of non‐CO_2_ greenhouse gases from the atmosphere*.

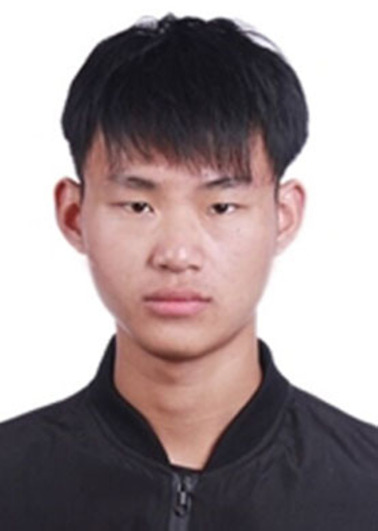

## Biographical Information

*Dr Yang Bai is a Senior Analytical Officer in the School of GeoScience at the University of Edinburgh. She studied chemical engineering at the Nanjing University of Technology and obtained B.Eng. in 2003 and Ph.D. in 2009. Her research interests are in the areas of methane emmissions and greenhouse gas removal*.

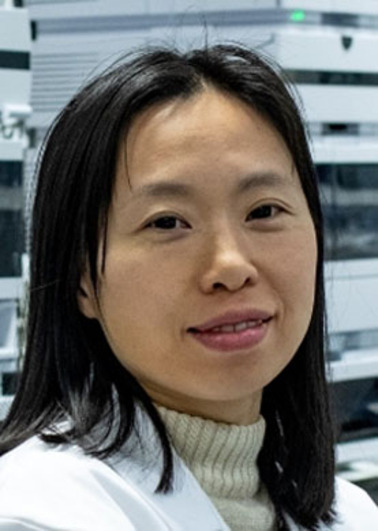

## Biographical Information

*Prof Xin Feng is a professor in Chemical Engineering at Nanjing Tech University. Ph.D. in Chemical Engineering at Nanjing University of Chemical Technology. She is mainly engaged in the preparation of potassium titanate whiskers and their application in composite materials, such as anti‐wear polymer composite material, long life high temperatures and pressures compressor oil‐free self‐lubricating seal element. She was awarded the “Moving Jiangsu Education Person ‐ Most Beautiful College Teacher in 2018 ” and the Second Prize of National Technological Invention Award in China (2009)*.

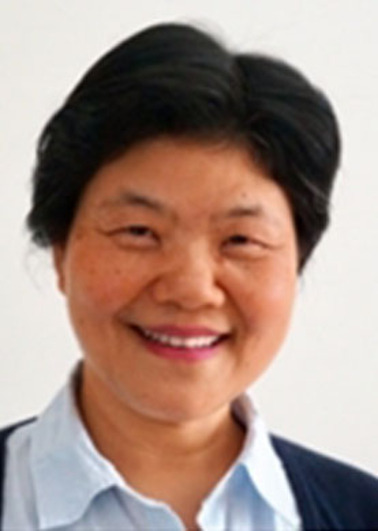

## Biographical Information

*Prof Jiahua Zhu is a professor of Chemical Engineering at Nanjing Tech University. His research interest covers the biomass processing, biomass‐based advanced materials and bio‐energy conversion by using advanced chemical technologies. Dr. Zhu has coauthored more than 160 peer‐reviewed journal articles and 4 book chapters. Dr. Zhu received Young Leader Development Award from Functional Material Division of The Minerals, Metals & Materials Society, Early Career Award from Polymer Processing Society and Early Career Investigator Award from ECS Electrodeposition Division*.

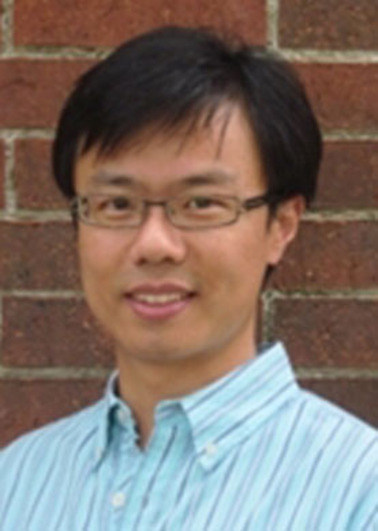

## Biographical Information

*Prof Xiaohua Lu is a professor in Chemical Engineering at Nanjing Tech University. He received his Ph.D. in Chemical Engineering from Nanjing University of Chemical Technology in 1988. He has been a leader in fundamental theoretical modelling and experimental measurements for complex fluids with interfaces. He also pioneered the extension of non‐equilibrium statistical mechanics to interfaces. Up to now, he has published 300+ journal articles*.

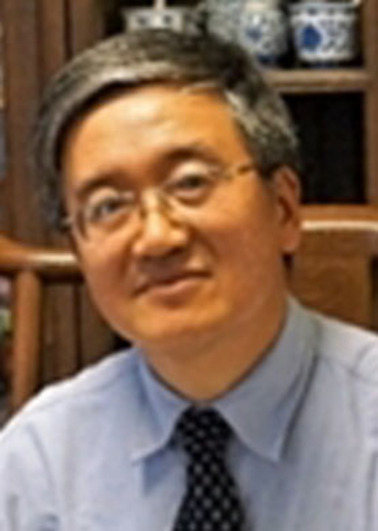

## Biographical Information

*Prof Liwen Mu is a professor of Chemical Engineering at Nanjing Tech University. He received his Ph.D. in Chemical Engineering at Nanjing Tech University in 2012. His current research focuses on conversion of non‐CO_2_ greenhouse gases, interfacial heat transfer and drag reduction, and high‐value utilization of biomass materials. He has co‐authored more than 80 peer‐reviewed journal articles and 1 book chapter*.

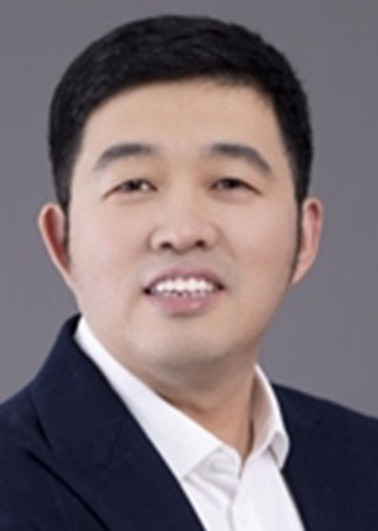

## Biographical Information

*Prof Tingzhen Ming, Chair Built Environment and Energy Engineering, School of Civil Engineering and Architecture, Wuhan University of Technology. Subject Editor of Journal of Thermal Science. His research interests are: CFD, heat and mass transfer, urban planning, building and environment, pollutant dispersion. Prior to join in WHUT, He worked as an associate professor at Huazhong University of Science and Technology. Prof. Ming published over 150 journal papers, 3 books in Elsevier Academic Press, Springer Press, and Science Press (China)*.

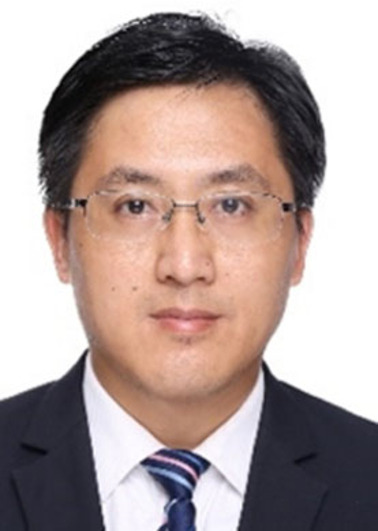

## Biographical Information

*Dr Renaud de Richter is a scientific advisor to Methane Action. He graduated from the organic chemistry department of the engineering school of chemistry of Montpellier, France in 1985 with a Master's degree in chemistry. Renaud received his PhD degree in 1989 from University of Science and Technology in Montpellier, France. His current research interests include the application of photocatalytic chemistry and processes for environmental protection to fight climate change and for remediation purposes*.

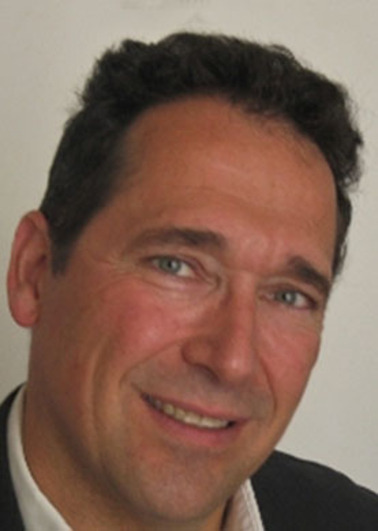

## Biographical Information

*Dr Wei Li joined the University of Edinburgh in 2021 as a Senior Lecturer in Chemical Engineering. He studied chemical engineering at the Nanjing University of Technology, obtaining his B.Eng. and Ph.D. in 2008. His first employment started at The University of Hong Kong (Department of Chemistry), followed by research positions at the Ludwig‐Maximilians‐Universität München (Department of Physics) and the University of Liverpool. Before he joined UoE, Dr Li also had worked at the National Graphene Institute, University of Manchester, and Aston University. His expertise includes nanoengineering of photocatalytic materials and reaction engineering of photocatalytic processes*.

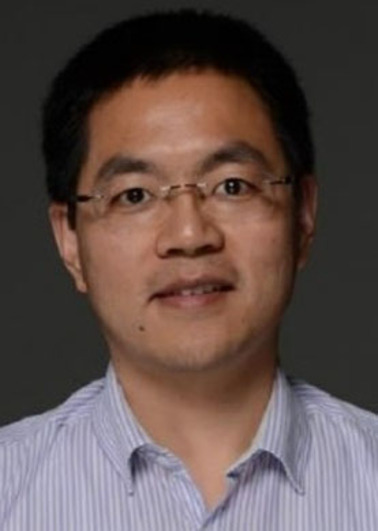

## Data Availability

The data that support the findings of this study are available from the corresponding author upon reasonable request.
